# A20-Binding Inhibitor of NF-*κ*B 1 Ameliorates Neuroinflammation and Mediates Antineuroinflammatory Effect of Electroacupuncture in Cerebral Ischemia/Reperfusion Rats

**DOI:** 10.1155/2020/6980398

**Published:** 2020-10-13

**Authors:** Xueling Zhou, Wenhao Lu, You Wang, Jiani Li, Yong Luo

**Affiliations:** ^1^Department of Neurology, The First Affiliated Hospital of Chongqing Medical University, Chongqing 400016, China; ^2^Laboratory Research Center, The First Affiliated Hospital of Chongqing Medical University, Chongqing 400016, China; ^3^Department of Neurology, The Second Affiliated Hospital of Chongqing Medical University, Chongqing 400010, China

## Abstract

A20-binding inhibitor of NF-*κ*B 1 (ABIN1) is an inhibitor of NF-*κ*B and exerts anti-inflammatory effect. Electroacupuncture (EA) is considered as a neuroprotective strategy by inhibiting neuroinflammatory damage after cerebral ischemia. This study was performed to explore the role of ABIN1 and investigate whether the ABIN1 is involved in the mechanism of EA in cerebral ischemia/reperfusion (I/R) rats. Male Sprague-Dawley (SD) rats were subjected to middle cerebral artery occlusion/reperfusion (MCAO/R) and received EA after reperfusion once a day. Lentivirus-mediated ABIN1 gene knockdown was used to detect the role of ABIN1 in neuroinflammation after I/R. ABIN1 expression, proinflammatory cytokine levels, microglial activation, neurological function, infarct volumes, and NF-*κ*B activation were assessed. ABIN1 expression was elevated in the peri-infarct cortex and was further upregulated by EA. ABIN1 knockdown increased the levels of proinflammatory cytokines and activation of microglia, worsened neurological deficits, and enlarged the infarct volume. Moreover, ABIN1 was blocked to partially reverse the neuroprotective effect of EA, and this treatment weakened the ability of EA to suppress NF-*κ*B activity. Based on these findings, ABIN1 is a potential suppressor of neuroinflammation and ABIN1 mediates the antineuroinflammatory effect of EA in cerebral I/R rats.

## 1. Introduction

Ischemic stroke accounts for 84.4% of all strokes, resulting in a high global burden [1]. With the development of endovascular therapy, the treatment of acute ischemic stroke has entered a new stage [2]. However, recanalization can lead to cerebral ischemia/reperfusion damage. Thus, neuroprotection combined with reperfusion therapy is an important next step in the development of ischemic stroke treatment [3, 4]. The pathological mechanism of cerebral ischemia/reperfusion (I/R) is complex. Excessive neuroinflammation after cerebral I/R is one of the main culprits for aggravating brain damage [5–7], which has been considered as a potential therapeutic target [4, 8]. NF-*κ*B acts as a “molecular switch” in ischemic stroke, promoting the initiation and amplification of the inflammatory cascade [9]. The most common dimeric form of NF-*κ*B is p65/p50, and the dimer binds to inhibitor of kappa B (I*κ*B) and is localized in the cytoplasm in the resting state. Upon stimulation by cerebral ischemia, I*κ*B proteins are phosphorylated by the inhibitor of kappa B kinase (IKK) and degraded, allowing NF-*κ*B to enter the nucleus and promote the expression of a series of proinflammatory cytokines [9–11].

A20-binding inhibitor of NF-*κ*B 1 (ABIN1) is a physiological inhibitor of NF-*κ*B that binds the Lys63 and Met-1 polyubiquitin chains with high affinity [12, 13]. As an adaptor protein of A20, ABIN1 promotes the deubiquitination of molecules upstream of NF-*κ*B and inhibits NF-*κ*B activation [14, 15]. ABIN1-deficient mice die during embryogenesis and the mice that survive exhibit immune cell activation and develop a progressive, lupus-like inflammatory disease [16, 17]. Notably, ABIN1 acts as an inhibitor of inflammation in various inflammatory diseases, such as hepatic I/R, asthma, systemic lupus erythematosus, psoriasis, and osteoarthritis [18–22]. However, the role of ABIN1 in neuroinflammatory damage after cerebral I/R has not been reported.

Electroacupuncture (EA) is a product of modern technology that adds electrical stimulation to acupuncture, which is characterized by a low cost, few side effects, and controllable parameters. EA regulates the immune response and restores homeostasis, which may underlie its use as a treatment for various inflammation-related diseases [23–25]. Furthermore, EA is a widely used treatment for ischemic stroke and has achieved good results [26–28]. The neuroprotective effect of EA is closely related to the inhibition of NF-*κ*B [29–31]; however, the specific regulatory mechanism of EA has not been completely clarified. As shown in our previous studies, EA inhibits I*κ*B*α* phosphorylation and prevents the nuclear translocation of NF-*κ*B p65 by upregulating the neuronal deubiquitinating enzyme A20 to ultimately improve the neurological deficits in middle cerebral artery occlusion/reperfusion (MCAO/R) rats [32]. Interestingly, ABIN1 is an important member of the A20 complex and may also be regulated by EA. Considering the fact that ABIN1 plays a crucial role in inhibiting NF-*κ*B-related inflammatory responses, EA may inhibit NF-*κ*B activation and exert neuroprotective effect via upregulating ABIN1 expression in cerebral I/R rats. Therefore, in the present study, we initially explored the effect of ABIN1 on neuroinflammatory damage in a MCAO/R rat. Then, the possible mechanism by which EA regulates the expression of ABIN1 to inhibit NF-*κ*B activation was investigated.

## 2. Methods

### 2.1. Animals

Healthy Sprague-Dawley (SD) rats weighing 280–300 g were purchased from Experimental Animal Center of Chongqing Medical University (Chongqing, China). Animals were maintained in a specific pathogen‐free (SPF) room (12 h light/dark cycle, 22 ± 2°C, 60–70% humidity). Rats were allowed for free access to food and water. Rats were kept for a week to acclimatize before the experiment. All animal experimental procedures were approved by the Ethics Committee for Animal Experimentation of Chongqing Medical University (number SYXK(Yu) 2018-0003) and were performed in accordance with National Institutes of Health Guide for the Care and Use of Laboratory Animals.

In the first study, rats were randomly divided into 4 groups: sham group, MCAO/R group, MCAO/R + EA group, and MCAO/R + sham EA group to investigate the expression of ABIN1 in the peri-infarct cortex.

The second study elucidated the effect of ABIN1 on neuroinflammatory damage after I/R using sham group, MCAO/R group, MCAO/R + LV-Scramble group, and MCAO/R + LV-shABIN1 group.

The third study explored whether ABIN1 was involved in the antineuroinflammatory mechanism of EA using MCAO/R group, MCAO/R + EA group, MCAO/R + EA + LV-Scramble group, and MCAO/R + EA + LV-shABIN1 group.

### 2.2. Establishment of the MCAO/R Model

A total of 208 rats were used in this experiment, including 17 rats that were excluded due to death (*n* = 14) or unsuccessful induction of ischemia (*n* = 3). SD rats underwent MCAO/R as previously described [33]. Briefly, rats were anesthetized with sodium pentobarbital (60 mg/kg, i.p.). A nylon monofilament with a silicon-coated rounded tip was inserted to block the right middle cerebral artery. After 2 h of ischemia, the nylon monofilament was removed to induce reperfusion. Rats were detected by a laser Doppler flowmetry (PeriFlux 5000, Perimed AB, Sweden), and regional cerebral blood flow decreased to 20% and recovered to >80% of baseline, indicating the successful establishment of the MCAO/R model. The rats in the sham group underwent the same operation, except that the monofilament was not inserted to block the middle cerebral artery. Rat rectal temperature was maintained at 37 ± 0.5°C with an electrothermal pad.

### 2.3. Intracerebral Lentivirus Injection

Lentiviruses containing the ABIN1 shRNA (LV-shABIN1) for ABIN1 knockdown and control shRNA (LV-Scramble) were supplied by Genechem (Shanghai, China). Two weeks prior to the establishment of the MCAO/R model, the right cortex of rat was injected with the lentiviruses. The injection sites were as follows: site 1, A-P 1.0 mm; M-L −2.0 mm; D-V −1.2 mm, and site 2, A-P −3.0 mm; M-L −1.5 mm; D-V −1.2 mm [34]. A total of 2.5 *μ*l of LV-shABIN1 or LV-Scramble were injected into each site.

### 2.4. EA Treatment

**Figure 1:**
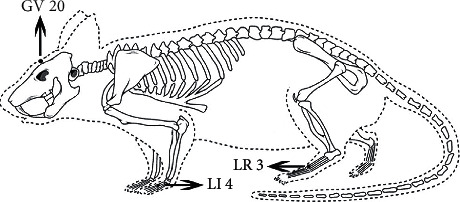
Schematic diagram of GV20, LI 4, and LR 3 acupoints of rat.

Immediately after reperfusion, the rat was treated with EA once a day until sacrifice. EA was performed as described previously [32] and acupuncture needles were inserted at Baihui (GV 20), Hegu (LI 4), and Taichong (LR 3) (Figure 1) and connected with EA instrument (Model no. SDZ-III, Hwato, China). To construct a circuit, two electrodes were connected, respectively, to the needle at GV 20 and the left ear (nonacupoint). Another two electrodes were connected, respectively, to the needle at LI 4 and LR 3. The stimulation parameters were intensity of 1 mA and a frequency of 20 Hz for 5 min followed by 2 Hz for 30 min. In the sham EA group, needles were affixed to the acupoints without skin penetration, and the animals did not receive electrical stimulation [35].

### 2.5. Evaluation of the Neurological Function

At 72 h before and 24, 48, and 72 h after cerebral I/R, each rat was assessed with the Modified Neurological Severity Score (mNSS) and Modified Sticky-Tape Test (MST) by an observer who was blinded to the experiments. The mNSS is a comprehensive scoring system that includes motor, balance, sensory, and reflex tests and is graded on a scale of 0 (normal) to 18 (maximal deficit) points [36]. The Modified Sticky-Tape Test was used to evaluate somatosensory dysfunction. The sticky paper tape (3 cm long and 1 cm wide) was placed around the paw of the rat, and the time for the rat to tear the tape within 30 seconds was recorded. Per limbs was tested 5 times a day, and Modified Sticky-Tape Test performance was presented as a ratio of left/right [37].

### 2.6. Measurement of the Cerebral Infarct Volume

Rats were euthanized with an overdose of sodium pentobarbital (150 mg/kg, i.p.). After sacrifice, brains were cut into 2 mm thick serial coronal slices and immersed in 2,3,5-triphenyltetrazolium chloride (TTC) at 37°C for 15 min in the dark. Images were acquired with a camera and analyzed with ImageJ software. The infarct volume was presented as a percentage of the intact hemisphere.

### 2.7. Real-Time Quantitative PCR (RT-qPCR)

**Figure 2:**
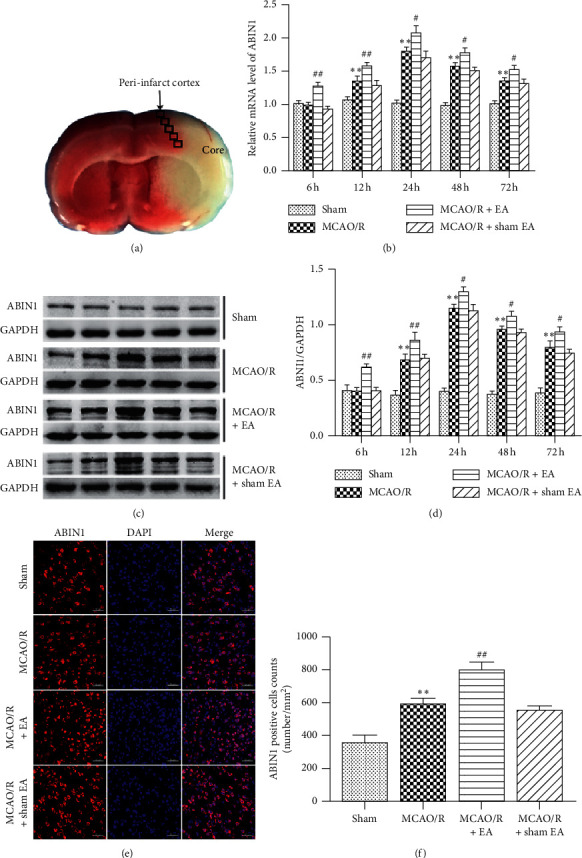
ABIN1 expression in the peri-infarct area at different time points. (a) The core and peri-infarct areas of a MCAO/R rat. (b–d) RT-qPCR and western blot were used to, respectively, detect the levels of the ABIN1 mRNA and protein in the peri-infarct cortex at 6 h, 12 h, 24 h, 48 h, and 72 h after reperfusion (*n* = 5 rats per group). The expression of ABIN1 was normalized to GAPDH. (e) ABIN1 (red) and DAPI (blue) immunofluorescence staining present the distribution of ABIN1 at 24 h after reperfusion (*n* = 3 rats per group). Scale bar = 50 *μ*m. (f) Column chart presenting the ABIN1^+^ cell counts in the four groups (^*∗*^*P* < 0.05 and ^*∗∗*^*P* < 0.01 compared to the sham group; #*P* < 0.05 and ^##^*P* < 0.01 compared to the MCAO/R group).

As shown in Figure 2(a), the pale area was considered to be the infarct core. Strips of tissue (2 mm thick) surrounding the infarct core were considered as the peri-infarct tissue and dissected for RT-PCR and western blot analysis [38]. Total RNA was extracted from the peri-infarct cortex using Trizol (TaKaRa, Japan). Then, cDNAs were synthesized using a PrimeScript RT reagent kit with gDNA Eraser (TaKaRa) and were used as a template for RT-qPCR, which was conducted in a CFX96 Real-Time PCR Detection System (Bio-Rad Co., USA) with SYBR Green. The following primer sequences were used (5′ to 3′): GAPDH: forward: AAGTTCAACGGCACAGTCAAGG and reverse: ACGCCAGTAGACTCCACGACAT, and ABIN1: forward: TCGGCTGAAGGGAAAAATACA and reverse: CAAAGGAGACCAAGGAGGGAG. The results were normalized to the levels of the housekeeping gene GAPDH.

### 2.8. Western Blot Analysis

Proteins were extracted from the peri-infarct cortex using RIPA buffer (Beyotime, China). A cytoplasmic/nuclear protein extraction kit (Beyotime) was used to extract cytoplasmic and nuclear proteins. Western blot was performed as described previously [39]. Briefly, proteins were separated on 10% gels and transferred to PVDF membranes. After blocking with 5% skim milk, membranes were incubated with the primary antibodies at 4°C overnight. The following primary antibodies against specific proteins were used: ABIN1 (#4664, Cell Signaling Technology, USA, 1 : 1000), A20 (#5630, Cell Signaling Technology, USA, 1 : 1000), NF-*κ*B p65 (#8242, Cell Signaling Technology, 1 : 1000), I*κ*B*α* (18220-1-AP, Proteintech, USA, 1 : 000), phospho-I*κ*B*α* (Ser32) (#2859, Cell Signaling Technology, 1 : 1000), GAPDH (10494-1-AP, Proteintech, USA, 1 : 1000), and histone H3 (#3638, Cell Signaling Technology, 1 : 1000). Membranes were washed with TBST and immersed in the secondary antibodies at 37°C for 1 h. After washes with TBST, immunoreactive bands were detected using WesternBright ECL (Advansta, USA). Membranes were scanned and analyzed using a Fusion FX5 analysis system (Vilber Lourmat Fusion FX 7 Spectra, France).

### 2.9. ELISA

The peri-infarct cortex was collected 24 h after reperfusion, and the concentrations of TNF-*α*, MCP-1, and IL-1*β* were assayed using ELISA kits (nos. EK0526, EK0902, and EK0393, respectively, BOSTER, China).

### 2.10. Immunofluorescence Staining

Brain tissues were fixed, dehydrated, and then cut into coronal sections at a thickness of 10 *μ*m. Sections were permeabilized with 0.3% Triton X-100. Then, brain slices were blocked with 5% donkey or goat serum for 1 h and incubated overnight at 4°C with primary antibodies: ABIN1 (bs-9568R, Bioss, China, 1 : 50), NeuN (MAB377, Millipore, Germany, 1 : 200), A20 (3A11G6, Proteintech, USA, 1 : 100), Iba-1 (NB100-1028, Novus, USA, 1 : 50), and GFAP (BM0055, Boster, China, 1 : 100). Then, the following fluorescently labeled secondary antibodies were incubated with the sections at 37°C for 1 h in the dark: CoraLite594-goat anti-rabbit (SA00013-4, Proteintech, 1 : 200), CoraLite488-goat anti-mouse (SA00013-1, Proteintech, 1 : 200), CoraLite594-donkey anti-rabbit (SA00013-8, Proteintech, 1 : 200), FITC-donkey anti-goat (SA00003-3, Proteintech, 1 : 200), and Cell nuclei were stained with DAPI. Brain slices were observed under a laser confocal microscope (LSM-800, Carl Zeiss Micro-Imaging Co., Germany).

### 2.11. Coimmunoprecipitation

The peri-infarct cortex was homogenized in IP lysis buffer (Beyotime, China) to extract the proteins. One microgram of anti-ABIN1 antibody (#4664, Cell Signaling Technology), anti-A20 antibody (#5630, Cell Signaling Technology), or normal rat IgG was added to each milligram of total protein in the supernatants, and the samples were rotated at 4°C overnight. Forty microliters of protein A/G agarose beads was mixed with the supernatants and rotated at 4°C for 2 h. The beads were washed with lysis buffer and then eluted with 40 *μ*l of SDS loading buffer. The supernatants were collected for western blot.

### 2.12. Statistical Analyses

All data were analyzed using SPSS 21.0 and graphed using GraphPad Prism 8.0 and expressed as the means ± SEMs. The differences of cellular localization of ABIN1 were assessed using the unpaired *t*-test. All other quantitative data were analyzed using one-way ANOVA. *P* < 0.05 was considered statistically significant.

## 3. Results

### 3.1. ABIN1 Expression Is Induced in MCAO/R Rats

We measured the levels of the ABIN1 mRNA and protein in the peri-infarct cortex at 6 h, 12 h, 24 h, 48 h, and 72 h after reperfusion. The expression of the ABIN1 mRNA and protein peaked in the MCAO/R group at 24 h after reperfusion and then gradually decreased (Figures 2(b)–2(d)). The ABIN1 mRNA and protein were expressed at higher levels in the MCAO/R group than in the sham group from 12 h to 72 h (Figures 2(b)–2(d)). Moreover, compared with the MCAO/R group, EA further increased the expression of the ABIN1 from 6 h to 72 h (Figures 2(b)–2(d)). In addition, ABIN1 was detected using immunofluorescence staining at 24 h after reperfusion (Figures 2(e) and 2(f)). Compared to the sham group, the number of ABIN1^+^ cells in the peri-infarct cortex was significantly increased in the MCAO/R group (Figures 2(e) and 2(f)). Moreover, the number of ABIN1^+^ cells was further increased in rats receiving EA (Figures 2(e) and 2(f)). A significant difference in ABIN1 expression was not observed between the MCAO/R group and MCAO/R + sham EA group (Figures 2(b)–2(f)).

### 3.2. The Distribution of ABIN1 in the Peri-Infarct Cortex

**Figure 3:**
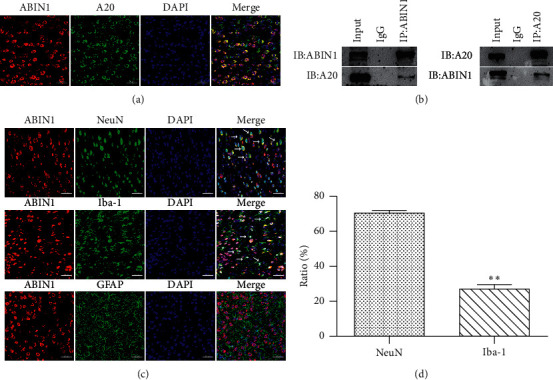
ABIN1 is colocated with A20 and NeuN and Iba-1, respectively, in the peri-infarct cortex. (a) Double immunofluorescence staining for ABIN1 and A20 in the peri-infarct cortex at 24 h after reperfusion (*n* = 3 rats per group). Scale bar = 50 *μ*m. (b) Coimmunoprecipitation of ABIN1 and A20 in the peri-infarct cortex at 24 h after reperfusion (*n* = 3 rats per group). (c) Double immunofluorescence labeling of ABIN1 (red) and NeuN (green, neurons), Iba-1 (green, microglia), and GFAP (green, astrocytes), respectively (*n* = 3 rats per group). White arrows show that ABIN1 is colocalized with NeuN and Iba-1, respectively. Scale bar = 50 *μ*m. (d) Comparisons of the percentage of ABIN1^+^ NeuN^+^ cells among ABIN1^+^ cells and ABIN1^+^ Iba-1^+^ cells among ABIN1^+^ cells in the peri-infarct cortex. ^*∗∗*^*P* < 0.01 compared to ABIN1^+^ NeuN^+^/ABIN1^+^.

ABIN1 has been described as an NF-*κ*B suppressor through interacting with A20 [12]; to further verify the interaction between A20 and ABIN1 in the peri-infarct cortex, the immunofluorescence staining and coimmunoprecipitation were performed. The immunofluorescence staining revealed numerous ABIN1^+^ and A20^+^ cells in the peri-infarct cortex 24 h after reperfusion, and A20 and ABIN1 were colocalized in the cytoplasm (Figure 3(a)). The coimmunoprecipitation results further suggested that ABIN1 and A20 bind to each other in the peri-infarct cortex at 24 h after reperfusion (Figure 3(b)).

Next, the cellular localization of ABIN1 in the peri-infarct cortex 24 h after reperfusion was evaluated using double immunofluorescence labeling. ABIN1 was colocalized with NeuN (a neuron marker) and Iba-1 (a microglia marker) but not with GFAP (an astrocyte marker) (Figure 3(c)). Moreover, ABIN1^+^NeuN^+^ cells accounted for 70.22 ± 1.71% of ABIN1^+^ cells, and ABIN1^+^Iba-1^+^ cells accounted for 26.83 ± 2.75 % of ABIN1^+^ cells in the peri-infarct cortex (Figures 3(c) and 3(d)). These results indicated that neurons and microglia were the cellular source of ABIN1 in the peri-infarct cortex.

### 3.3. ABIN1 Knockdown Increases Proinflammatory Cytokines Production and Microglial Activation

**Figure 4:**
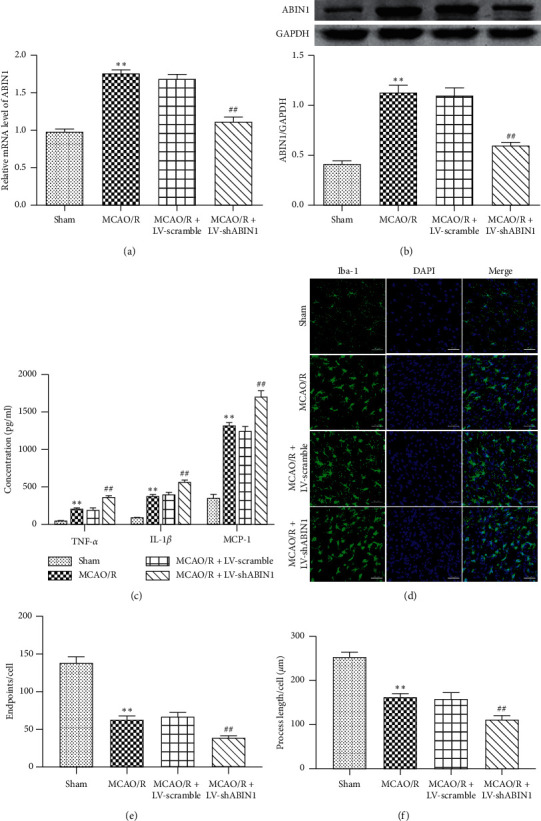
ABIN1 knockdown increases proinflammatory cytokine production and microglial activation. (a-b) Levels of the ABIN1 mRNA and protein were detected 24 h after reperfusion using RT-qPCR and western blot, respectively, to confirm the efficiency of ABIN1 gene knockdown (*n* = 5 rats per group). (c) ELISA was used to detect the concentrations of TNF-*α*, IL-1*β* and MCP-1 in the peri-infarct cortex at 24 h after reperfusion (*n* = 5 rats per group). (d) Microglial morphology was observed using immunofluorescence staining for Iba-1 (green) in the peri-infarct cortex at 24 h after reperfusion (*n* = 3 rats per group). Scale bar = 50 *μ*m. (e and f) Quantification of microglia process endpoints and cell and process lengths/cell (^*∗∗*^*P* < 0.01 compared to the sham group; ^##^*P* < 0.01 compared to the MCAO/R group).

ABIN1 was silenced by a cortical injection of LV-shABIN1 to further explore the role of ABIN1 in neuroinflammation after I/R. First, the levels of the ABIN1 mRNA and protein were measured at 24 h after reperfusion to ensure the effectiveness of ABIN1 knockdown. The expression of ABIN1 was significantly reduced in the MCAO/R + LV-shABIN1 group (Figures 4(a) and 4(b)), indicating that ABIN1 was successfully silenced.

Then, we examined the production of proinflammatory factors and microglial activation using ELISA and immunofluorescence staining, respectively. Typical proinflammatory factors TNF-*α*, IL-1*β,* and MCP-1 were produced at higher levels in the MCAO/R group than in the sham group (Figure 4(c)). Higher levels of these cytokines were detected in the MCAO/R + LV-shABIN1 group than in the MCAO/R group (Figure 4(c)). The levels of these proinflammatory factors were not significantly different between the MCAO/R + LV-scramble group and the MCAO/R group (Figure 4(c)).

The morphology of microglia is closely related to their biological functions. Resting microglia are hyperramified. Activated microglia are characterized by enlarged cell bodies with short and thick processes and round and rod-like cell bodies, and some even exhibit an amoeba-like cell bodies [40]. We evaluated the activation of microglia by quantifying the number of endpoints per cell and the length of cell processes, which may serve as indicators of neuroinflammation [41, 42]. Immunofluorescence staining for Iba-1 was used to detect microglia in the peri-infarct cortex at 24 h after I/R. The microglia in the sham group exhibited the resting state phenotype (hyperramification) (Figure 4(d)). As shown in Figures 4(d)–4(f), the number of endpoints per cell and the length of cell process were decreased in the MCAO/R group compared with the sham group, indicating that more activated microglia were present in the MCAO/R rats. Moreover, the number of endpoints per cell and the length of cell process were reduced in the MCAO/R + LV-shABIN1 group compared with the MCAO/R group, suggesting that blockade of ABIN1 expression enhanced microglial activation (Figures 4(d)–4(f)). Significant differences were not observed between the MCAO/R + LV-scramble group and the MCAO/R group (Figures 4(d)–4(f)).

### 3.4. ABIN1 Knockdown Exacerbates the Neurological Deficits and Enlarges the Infarct Volume

**Figure 5:**
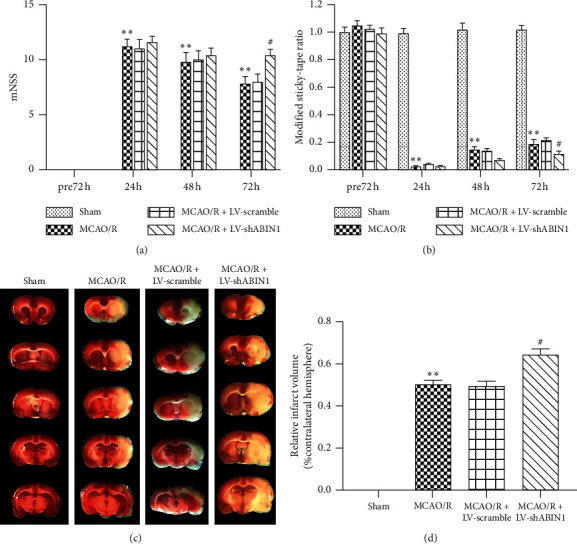
ABIN1 knockdown exacerbates the neurological deficits and enlarges the infarct volume. (a and b) The mNSS and MST were, respectively, analyzed at 72 h before and 24, 48, and 72 h after reperfusion (*n* = 5 rats per group). (c) Brain tissue sections were stained with TTC at 72 h after reperfusion (*n* = 5 rats per group). (d) The infarct volume is presented as a percentage of the intact hemisphere (^*∗∗*^*P* < 0.01 compared to the sham group; ^#^*P* < 0.05 compared to the MCAO/R group).

The mNSS and MST were used to assess the neurological function of rats 72 h before and 24, 48, and 72 h after cerebral I/R. At 72 h before reperfusion, the neurological function of rats was normal and no significant differences were observed among the groups (Figures 5(a) and 5(b)). The mNSS and MST ratio of the MCAO/R group were worse than the sham group at 24, 48, and 72 h after I/R (Figures 5(a) and 5(b)). Compared with the MCAO/R group, the neurological deficits of the MCAO/R + LV-shABIN1 group were increased significantly at 72 h but not at 48 h or 24 h (Figures 5(a) and 5(b)). The neurological deficits of the MCAO/R + LV-scramble group was not different from the MCAO/R group at 24, 48, and 72 h after I/R (Figures 5(a) and 5(b)). The infarct volume was assessed using TTC staining at 72 h after reperfusion. The cerebral infarct volume did not differ significantly between the MCAO/R + LV-scramble group and the MCAO/R group but was expanded in the MCAO/R + LV-shABIN1 group (Figures 5(c) and 5(d)).

### 3.5. ABIN1 Knockdown Weakens the Antineuroinflammatory Effect of EA to Some Extent

**Figure 6:**
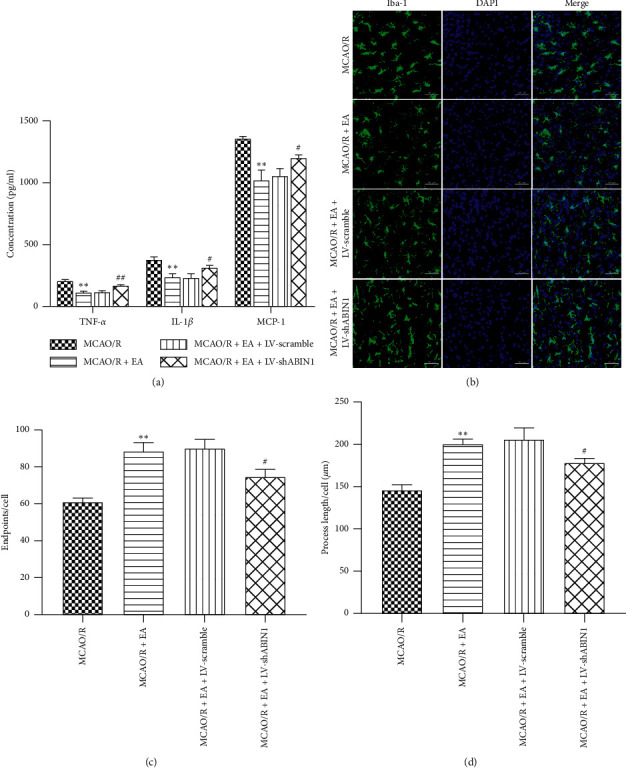
ABIN1 knockdown impairs the antineuroinflammatory effect of EA. (a) The concentrations of TNF-*α*, IL-1*β,* and MCP-1 in the peri-infarct cortex were detected using ELISAs at 24 h after reperfusion (*n* = 5 rats per group). (b) Microglial morphology was observed by conducting immunofluorescence staining for Iba-1 (green) in the peri-infarct cortex at 24 h after reperfusion (*n* = 3 rats per group). Scale bar = 50 *μ*m. (c and d) Quantification of microglial endpoints/cell and process length/cell (^*∗∗*^*P* < 0.01 compared to the MCAO/R group; ^#^*P* < 0.05 and ^##^*P* < 0.01 compared to the MCAO/R + EA group).

The concentrations of TNF-*α*, IL-1*β,* and MCP-1 in the peri-infarct cortex were detected at 24 h after reperfusion using ELISA to determine the effect of ABIN1 knockdown on the antineuroinflammatory effect of EA. The levels of these proinflammatory cytokines were significantly decreased in the MCAO/R + EA group compared with the MCAO/R group (Figure 6(a)). In addition, ABIN1 knockdown impaired the antineuroinflammatory effect of EA, since higher levels of TNF-*α*, IL-1*β,* and MCP-1 were detected in the MCAO/R + EA + LV-shABIN1 group than in the MCAO/R + EA group (Figure 6(a)). The MCAO/R + EA + LV-scramble and MCAO/R + EA groups displayed similar levels of these cytokines (Figure 6(a)). Furthermore, the results of Iba-1 immunofluorescence staining in the peri-infarct cortex at 24 h after reperfusion indicated that the MCAO/R + EA group had more endpoints per cell and longer cell processes than the MCAO/R group; however, ABIN1 knockdown in the MCAO/R + EA + LV-shABIN1 group promoted microglial activation (Figures 6(b)–6(d)). Microglia activation was not significantly different between the MCAO/R + EA + LV-scramble group and MCAO/R + EA group (Figures 6(b)–6(d)).

### 3.6. ABIN1 Knockdown Partially Inhibits the Neuroprotective Effect of EA

**Figure 7:**
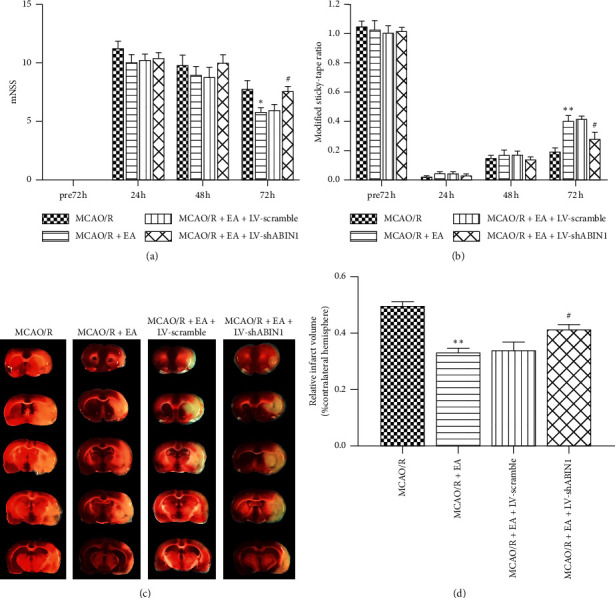
ABIN1 knockdown inhibits the neuroprotective effect of EA. (a-b) The mNSS and MST were recorded at 72 h before and 24, 48, and 72 h after reperfusion (*n* = 5 rats per group). (c) Brain tissue sections were stained with TTC at 72 h after reperfusion (*n* = 5 rats per group). (d) The infarct volume is presented as a percentage of the intact hemisphere (^*∗*^*P* < 0.05 and ^*∗∗*^*P* < 0.01 compared to the MCAO/R group; ^#^*P* < 0.05 compared to the MCAO/R + EA group).

The mNSS and MST of rats in the four groups were assessed at 72 h before and 24, 48, and 72 h after reperfusion. At 72 h before reperfusion, the neurological function of rats was normal and no significant differences were observed among groups. The neurological impairments observed in rats in the MCAO/R + EA group were significantly improved at 72 h but not 24 and 48 h after reperfusion compared with rats in the MCAO/R group (Figures 7(a) and 7(b)). Rats in the MCAO/R + EA + LV-shABIN1 group exhibited worse neurological deficits than rats in the MCAO/R + EA group at 72 h after reperfusion (Figures 7(a) and 7(b)). The mNSS and MST of rats in the MCAO/R + EA group were similar to rats in the MCAO/R + EA + LV-scramble group (Figures 7(a) and 7(b)). TTC staining at 72 h after reperfusion revealed a smaller infarct volume in the MCAO/R + EA group than in the MCAO/R group (Figures 7(c) and 7(d)). However, in the MCAO/R + EA + LV-shABIN1 group, ABIN1 knockdown increased the infarct volume compared with the MCAO/R + EA group (Figures 7(c) and 7(d)). The infarct volume did not significantly differ between the MCAO/R + EA + LV-Scramble group and the MCAO/R + EA group (Figures 7(c) and 7(d)). Based on these results, EA exerted a protective effect on the brain after I/R, but ABIN1 knockdown weakened the effect of EA.

### 3.7. EA Inhibits NF-*κ*B Activation by Upregulating ABIN1

**Figure 8:**
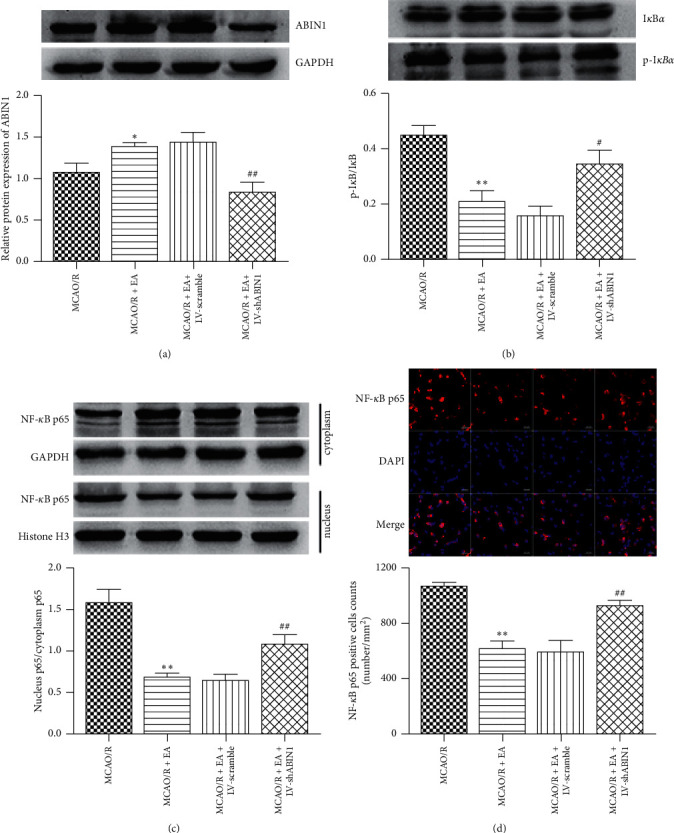
EA prevents NF-*κ*B activation by upregulating ABIN1 expression. (a-c) The levels of ABIN1, p-I*κ*B*α*, I*κ*B*α*, nuclear NF-*κ*B p65, and cytoplasmic NF-*κ*B p65 proteins in the peri-infarct cortex at 24 h after reperfusion were detected using western blot. GAPDH served as the internal reference for total and cytoplasmic proteins, and histone H3 served as the internal reference for nuclear proteins (*n* = 5 rats per group). (d) The nuclear translocation of NF-*κ*B p65 was observed with immunofluorescence staining in the peri-infarct area at 24 h after reperfusion (*n* = 3 rats per group). Scale bar = 20 *μ*m. Column chart presenting the NF-*κ*B p65^+^ cell counts in the four groups (^*∗*^*P* < 0.05 and ^*∗∗*^*P* < 0.01 compared to the MCAO/R group; ^#^*P* < 0.05 and ^##^*P* < 0.01 compared to the MCAO/R + EA group).

As mentioned above, NF-*κ*B plays a key role in focal cerebral I/R-induced neuroinflammation. To further explore whether ABIN1 mediated EA induced inhibition of NF-*κ*B activation, levels of the ABIN1, p-I*κ*B*α*, I*κ*B*α*, and nuclear/cytoplasmic NF-*κ*B p65 proteins were measured in the peri-infarct cortex at 24 h after reperfusion using western blot and the nuclear translocation of NF-*κ*B p65 was observed using immunofluorescence staining. EA significantly increased the level of the ABIN1 protein and decreased the p-I*κ*B*α*/I*κ*B*α* ratio and NF-*κ*B p65 nuclear translocation compared with the MCAO/R group (Figures 8(a)–8(d)). However, ABIN1 knockdown diminished the anti-inflammatory effect of EA as observed that p-I*κ*B*α*/I*κ*B*α* ratio was increased and NF-*κ*B p65 nuclear translocation was enhanced in the MCAO/R + EA + LV-shABIN1 group compared with the MCAO/R + EA group (Figures 8(a)–8(d)). The levels of these proteins were not significantly different between the MCAO/R + EA + LV-scramble group and the MCAO/R + EA group (Figures 8(a)–8(d)). Hence, EA may inhibit NF-*κ*B activation by upregulating ABIN1 expression.

## 4. Discussion

EA plays a beneficial role in ischemic stroke, but the mechanism of EA needs further research [43]. In the present study, we showed for the first time that ABIN1 was induced in MCAO/R rats. ABIN1 knockdown aggravated cerebral I/R injury by promoting activation of microglia and releasing proinflammatory factors (TNF-*α*, IL-1*β*, and MCP-1). Moreover, upregulation of ABIN1 expression was essential for EA to inhibit NF-*κ*B related neuroinflammatory damage after cerebral I/R.

ABIN1 mRNA is highly expressed in human peripheral lymphocytes, skeletal muscle, and spleen, and its polymorphisms are associated with autoimmune diseases [15]. We assessed the expression of ABIN1 in the early phase of cerebral I/R. The ABIN1 mRNA and protein were expressed in the cerebral cortex of the sham group, indicating that ABIN1 is constitutively expressed in the cerebral cortex under normal conditions. In addition, ABIN1 expression was induced in the peri-infarct cortex, peaking at 24 h and then gradually decreasing. NF-*κ*B is strongly activated during cerebral ischemia in cells such as neurons, microglia, astrocytes, and endothelial cells [11, 44]. The expression of some negative regulators of NF-*κ*B is regulated by NF-*κ*B to prevent its continuous activation [13, 45]. A20 is one of these negative regulators. A20 is expressed at low levels in most cells under physiological conditions, but its expression is rapidly induced upon the activation of NF-*κ*B [46]. Similarly, ABIN1 expression is also regulated by NF-*κ*B. The human ABIN1 gene promoter contains an NF-*κ*B response element, and its promoter activity is regulated by NF-*κ*B [47]. The expression of the ABIN1 mRNA is upregulated in various cell types with NF-*κ*B activation [15]. Thus, the increase in ABIN1 expression observed in the early stage of focal cerebral I/R may be attributed to the activation of NF-*κ*B, thus forming a negative feedback loop.

The A20 complex is expressed at higher levels in neurons than in glial cells [45, 48]. Similarly, as shown in our previous study, A20 is mainly expressed in neurons in the cortex of MCAO/R rats [32]. In the current study, we found that ABIN1 and A20 colocalized in the cytoplasm and interacted with each other in the peri-infarct cortex at 24 h. Besides, we determined the spatial distribution of ABIN1 in the peri-infarct cortex in MCAO/R rats for the first time. ABIN1 was prominently localized in neurons, followed by microglia, and no expression was observed in astrocytes at 24 h after I/R. The mechanism underlying the predominant expression of ABIN1 in neurons is unclear, but this differential distribution may be a protective strategy for neurons due to their high sensitivity to ischemia and hypoxia and limited tolerance to excessive activation of NF-*κ*B and neuroinflammation after I/R [49].

In the early phase of cerebral I/R, damaged neurons release cytokines, chemokines and damage-associated molecular patterns; microglia, the first line of defense in the brain, are activated within minutes after receiving signals from neurons [50, 51]. In the penumbra, proinflammatory microglia gradually dominate the response, resulting in an imbalance between proinflammatory and anti-inflammatory effects [52, 53]. Excessive proinflammatory factors such as TNF-*α*, IL-1*β,* and MCP-1 contribute to neuron death and the aggravation of brain damage [6, 54]. ABIN1 knockdown used lentivirus-mediated delivery of shABIN1 to investigate the effect of ABIN1 on focal cerebral I/R-induced neuroinflammation. ABIN1 knockdown increased microglial activation and proinflammatory factor production (TNF-*α*, IL-1*β*, and MCP-1), worsened neurological function, and enlarged the infarct volume. Thus, ABIN1 may confer neuroprotection by reducing inflammatory damage during cerebral I/R. However, in the MCAO/R group, the upregulation of endogenous ABIN1 expression by focal cerebral I/R is insufficient to resist strong neuroinflammation; thus, the increase in ABIN1 expression may represent a promising therapeutic strategy to alleviate neuroinflammation after cerebral I/R.

EA is a supplemental and alternative treatment for ischemic stroke that is recommended by the World Health Organization [26]. EA therapy requires a combination of specific acupoints and electrical stimulation. Acupoints GV 20, LI 4, and LR 3 exert the effects of tranquilization and resuscitation and are selected for treating ischemic stroke in traditional Chinese medicine [55, 56]. Our previous study showed that EA treatment at acupoints GV 20, LI 4, and LR 3 played a neuroprotective role in MCAO/R rats by suppressing NF-*κ*B activation [31]. Subsequently, it was shown that deubiquitinating enzyme A20 was upregulated by EA to inhibit I*κ*B*α* phosphorylation and prevent NF-*κ*B p65 nuclear translocation [32]. As an adaptor protein of A20, ABIN1 has similar biological functions to A20. For example, A20- and ABIN1-deficient mice showed premature death and severe inflammation, and the SNPs of A20 and ABIN1 genes were closely related to autoimmune diseases [12, 13]. Thus, whether ABIN1 is involved in the mechanism of EA is of great interest to us. The current study revealed that EA treatment at acupoints GV 20, LI 4, and LR 3 increased the expression of ABIN1 in the peri-infarct cortex. Since sham EA is biologically inactive because of the absence of important EA elements, such as the insertion of needles into acupoints and electrical stimulation [57–60], the expression of ABIN1 in peri-infarct cortex was not altered by sham EA. Besides, we showed that EA inhibited I*κ*B*α* phosphorylation, prevented NF-*κ*B p65 nuclear translocation, suppressed neuroinflammation, and improved neurological deficits. However, the neuroprotective effect of EA was partially reversed by ABIN1 knockdown. Based on these results, it was suggested that ABIN1 was involved in the mechanism of EA in alleviating cerebral I/R inflammatory damage. As mentioned above, ABIN1 was predominantly localized in neurons in the peri-infarct cortex; thus EA may inhibit neuronal NF-*κ*B activation by upregulating ABIN1 expression and eventually indirectly inhibit microglia aggregation and activation. Since some ABIN are also expressed in microglia, EA may reduce the production of proinflammatory factors by directly inhibiting NF-*κ*B activation in microglia. Further research will address these hypotheses in vitro to determine the effect of ABIN1 expression levels on NF-*κ*B activation in neurons and microglia, respectively.

## 5. Conclusion

In conclusion, ABIN1, which is induced in cerebral I/R, plays a neuroprotective role as an inflammatory suppressor. Furthermore, this study indicated that the EA-induced upregulation of ABIN1 expression may be an important mechanism by which EA blocks NF-*κ*B activation to alleviate neuroinflammation after cerebral I/R.

## Data Availability

The data used to support the findings of this study are available from the corresponding author upon request.
